# Heat stability of the in vitro inhibitory effect of spices on lipase, amylase, and glucosidase enzymes

**DOI:** 10.1002/fsn3.797

**Published:** 2019-01-28

**Authors:** Irushika T. Fernando, Kumudu I. Perera, Senarath B. P. Athauda, Ramiah Sivakanesan, Nimal Savitri Kumar, Lalith Jayasinghe

**Affiliations:** ^1^ Faculty of Medicine Department of Biochemistry University of Peradeniya Peradeniya Sri Lanka; ^2^ National Institute of Fundamental Studies Kandy Sri Lanka

**Keywords:** α‐amylase inhibition, boiling, glucosidase inhibition, lipase inhibition, Spices

## Abstract

This study investigated the effect of boiling on the inhibitory action of spices on digestive enzymes. Unboiled extracts of *Trigonella foenum‐graecum* (seed) (25.42%), *Myristica fragrans* (seed) (22.70%), and *Cuminum cyminum* (seed) (19.17%) showed significantly (*p *<* *0.05) a higher lipase inhibitory activity than their respective boiled extracts (20.23%, 15.74%, and 12.57%). Unboiled extracts of *Cinnamomum zeylanicum* (stem bark) (−16.98%) and *Foeniculum officinale* (seed) (−16.05%) showed an activation of lipase enzyme, and boiling significantly (*p *<* *0.05) changed the activity into lipase inhibition as 8.47% and 9.54%, respectively. Unboiled extracts of *Coriandrm sativum* (seed)*, C. cyminum*, and *Elettaria cardamomum* (seed) showed an activation of amylase enzyme, and boiling these extracts significantly reduced the enzyme activation. The other unboiled extracts showed a higher amylase inhibition than the boiled extracts, whereas the boiled extracts of *C.longa* (rhizome) and *Syzygium aromaticum* (flower) exhibited significantly (*p *<* *0.05) lower values. Unboiled extracts of *C. zeylanicum*,* M. fragrans*, and *S. aromaticum* showed an insignificantly higher glucosidase inhibitory activity than the boiled extracts. Inhibition of digestive enzymes by nutritional intervention is one avenue to be considered in treating diet‐induced obesity and in the management of postprandial hyperglycemia. Spices, used as food additives, could be a potential source of digestive enzyme inhibitors. The current study revealed that unboiled extracts of *T. foenum‐graecum* (seed), *C. cyminum* (seed), and *M. fragrans* (seed) are more effective than the boiled extracts as an antiobesity therapy. Moreover, it endorses the use of infusion of *T. foenum‐graecum* seeds as an antiobesity therapy.

## 1 INTRODUCTION

Herbs and spices have been used for thousands of years by many ancient cultures for curing ailments and for the promotion of good health (Kochhar, 2008). Dietary spices, used as flavoring and coloring agents and as preservatives, are obtained from the dried aroma parts of plants—generally seeds, berries, roots, pods, and leaves. Spices such as cinnamon, pepper, cloves, nutmeg, cumin, saffron, garlic, and ginger have been used in Asia and the Middle East for many centuries. Sri Lanka is well known for a variety of spices such as cinnamon, pepper, cloves, cardamoms, nutmeg, mace, and vanilla that grow in abundance all over the island in different types of soil and climatic conditions. These spices are an important part of the agricultural exports of Sri Lanka.

Obesity has become an epidemic increasing at an alarming rate (Katulanda, Jayawardena, Sheriff, Constantine, & Matthews, 2010; WHO; 2014) and is a major risk factor contributing to chronic diseases such as type 2 diabetes, cardiovascular diseases, and certain cancers (Jacobson, Miller, & Schaefer, 2007; Kopelman, 2000). Pancreatic lipase, which hydrolyzes triglycerides into glycerol and fatty acids, is the key enzyme for dietary fat digestion in the small intestine (Mukherjee & Sengupta, 2013). An inhibitor of pancreatic lipase can reduce fat absorption and can be used as a therapeutic agent for treating diet‐induced obesity in humans. Orlistat is the only clinically approved pharmacological agent used as a pancreatic lipase inhibitor (Weibel, Hadvary, Houchuli, Kupfer, & Lengsfeld, 1987).

Inhibition of carbohydrate hydrolyzing enzymes such as α‐glucosidase and pancreatic α‐amylase is one of the therapeutic approaches for delaying carbohydrate digestion, resulting in reduced postprandial hyperglycemia which is critical in the management of diabetes mellitus (Sudhir & Mohan, 2002). α‐Amylase catalyzes the hydrolysis of α‐1,4‐glucosidic linkages in starch and related polysaccharides. α‐Glucosidase secreted from intestinal epithelium is responsible for the degradation of oligosaccharides, trisaccharides, and disaccharides into monosaccharides. Inhibition of amylase and glucosidase enzymes would delay carbohydrate digestion and glucose absorption and thereby reduce postprandial hyperglycemia. Drugs that target carbohydrate hydrolyzing enzymes include acarbose, miglitol, voglibose, nojirimycin, and 1‐deoxynojirimycin, which reduce postprandial hyperglycemia by delaying glucose absorption (Bischoff, 1994).

The synthetic inhibitors of digestive enzymes lead to several adverse events in the gastrointestinal tract (Chiasson et al., 2002; Kaila & Raman, 2008). In this context, spices are an attractive source for the identification of newer digestive enzyme inhibitors that lack some of the adverse reactions of these synthetic enzyme inhibitors. Spices are usually used as food additives and in many recipes worldwide “cooking, baking, and roasting were applied to the spices.” Digestive enzyme inhibitors belonging to a variety of secondary metabolite groups such as polyphenols (flavonoids, phenolic acids, and proanthocyanidins) (Karamać & Amarowicz, 1996; Kim, Kwon, & Son, 2000; Shimura et al., 1994; You, Chen, Wang, Luo, & Jiang, 2011) and saponins (Zhao & Kim, 2004) have been identified, and these metabolites have been isolated in spices as well (Villupanoor, Chempakam, & Zachariah, 2008).

Therefore, the heat stability of inhibitors is vital for the inhibitory action to persist after cooking (Adefegha, Oboh, Oyeleye, & Osumo, 2016). The heat stability of the lipase, amylase, and glucosidase inhibitors of spices after processing (cooking at 100°C) has not been clearly investigated. Therefore, the investigation of the effects of boiling of spices on the lipase, amylase, and glucosidase inhibitory activities is important.

Hence, this study was designed to determine the lipase inhibitory activity, amylase inhibitory activity, and glucosidase inhibitory activity of unboiled and boiled crude methanol extracts of ten popular spices in Sri Lanka. Moreover, the heat stability of antioxidants of these spices was determined. The real‐time lipase inhibition assay used in this study was also optimized using the lipase assay developed by Choi et al. in 2003.

## 2 MATERIALS AND METHODS

### 2.1 Chemicals and reagents

Porcine pancreatic lipase (E.C.3.1.1.3, Type II) (L‐0382), porcine pancreatic α‐amylase (EC 3.2.1.1, Type VI) (A3176), α‐glucosidase from *Saccharomyces cerevisiae* (EC 3.2.1.20, Type I) (9001‐41‐7), 2,3‐dimercapto‐1‐propanol tributyrate substrate (DMPTB, 97%) (282413), 5,5‐dithiobis (2‐nitrobenzoic acid) (DTNB, Ellman's reagent) (D218200), 3,5‐dinitrosalicylic acid (128848), *p*‐nitrophenyl *α*‐D‐glucopyranoside (*p*NPG) (N‐1377), orlistat (O4139), acarbose (A8980), and 2,2‐diphenyl‐l‐picrylhydrazyl (DPPH) (D9132) were purchased from Sigma‐Aldrich, USA. Phosphate‐buffered saline (PBS) (003002), Tris‐HCl buffer (15504020), butylated hydroxyl anisole (BHA) (817023), and all the other chemicals used in this study were of analytical grade.

### 2.2 Plant collection and extraction

*Brassica juncea*,* Cinnamomum zeylanicum, Coriandrm sativum*,* Cuminum cyminum*,* Curcuma longa*,* Elettaria cardamomum*,* Foeniculum officinale*,* Myristica fragrans*,* Syzygium aromaticum*, and *Trigonella foenum‐graecum*, were purchased from a traditional drug store in Kandy, Sri Lanka. Voucher specimens were deposited at the Department of Biochemistry, Faculty of Medicine, University of Peradeniya, Sri Lanka. All the plant materials (Table 1) were washed with water and air‐dried in the shade. Dried and powdered plant material (100 g) was successively extracted using an ultrasonicator (VWR ultrasound cleaner, model‐USC 1700D—USA) with methanol (MeOH, 100 ml × 3). The extracts were evaporated to dryness using a rotary evaporator (Büchi Rotvapor^®^ R II—Switzerland) under reduced pressure at 40°C and kept in a vacuum oven at room temperature.

**Table 1:** Percentage yield (%) of crude methanol extracts of spices

Scientific name	Local name	Plant part used	Yield (%W/W)^a^
*Brassica juncea*	Aba	Seed	3.5
*Cinnamomum zeylanicum*	Kurundu	Stem bark	3.0
*Coriandrum sativum *	Kottamalli	Seed	2.7
*Cuminum cyminum*	Suduru	Seed	5.7
*Curcuma longa*	Kaha	Rhizome	8.0
*Elettaria cardamomum*	Ensal	Seed	2.1
*Foeniculum officinalis*	Ma'duru	Seed	4.8
*Myristica fragrans*	Sadikka	Seed	6.8
*Syzygium Aromaticum*	Karabu	Flower	4.7
*Trigonella foenum‐graecum*	Ulu‐hal	Seed	8.4

a Yield (%) of MeOH extracts of dry weight of plant material. Percentage extract yield (w/w) was calculated as (dry extract weight/dry starting material weight × 100).

### 2.3 Lipase inhibition assay

Lipase inhibition assay used during the current study was adapted from the lipase assay reported by Choi, Hwang, and Kim (2003). In this assay, free thiol groups that are generated by the lipase hydrolysis of DMPTB reduce DTNB to create a yellow color, which is spectrophotometrically quantified. The substrate mixture contained 0.2 mM DMPTB in 50 mM Tris‐HCl, pH 7.2, 0.001% EDTA, 0.06% Triton X‐100, and 0.8 mM DTNB. The porcine pancreatic lipase was prepared as a stock solution at 50 mg/ml in 50 mM Tris‐HCl, pH 7.2, and bovine serum albumin (BSA; 1 mg/ml), and stored at *−*40°C.

Optimization of lipase inhibition assay was conducted by evaluating the linearity of the method. Different reaction mixtures were prepared with different substrate concentrations (0.004 mM, 0.008 mM, and 0.016 mM), incubated with lipase (8 U) at 37°C, and the absorbances were recorded for 30 min at 412 nm.

For the screening of the extracts, porcine pancreatic lipase (8 U/ml) and plant extract (1 mg/ml; Tris‐HCl buffer or 4% v/v DMSO) were preincubated at 37°C. After preincubation, DMPTB (0.008 mM) standard substrate mixture was added to all tubes and incubated for 15 min. The absorbances were recorded after 15 min at 412 nm. Orlistat was used as the control/standard inhibitor.

The percentage lipase inhibition was calculated by the following formula;

$$\%\;\text{Lipase inhibition activity}=\frac{\text{Absorbance of Control}-\text{Absorbance of test}}{\text{Absorbance of Control}}\times 100$$

### 2.4 Amylase inhibition assay

The amylase inhibition assay was performed using the preincubation chromogenic method adapted from Geethalakshmi, Sarada, Marimuthu, and Ramasamy (2010). The plant extract (40 μl, 20 mg/ml in dimethylsulfoxide, DMSO), 160 μl of distilled water, was preincubated with the addition of 200 μl of the enzyme solution for 5 min at 37°C before reacting with the starch solution (400 μl) for 15 min. The mixture (200 μL) was removed and added into a separate tube containing 100 μL 3,5‐dinitrosalicylic acid (DNS) color reagent solution and placed in a 85°C water bath. After 15 min, this mixture was diluted with 900 μl distilled water and removed from the water bath. α‐Amylase activity was determined by measuring the absorbance of the mixture at 540 nm. Acarbose was used as the control/standard inhibitor.

Percentage maltose generated was calculated from the equation obtained from the maltose standard calibration curve (0%–0.2%, w/v maltose)

% reaction = (mean maltose in test/mean maltose in control) × 100.

% Amylase inhibition activity = (100−% reaction)

### 2.5 Glucosidase inhibition assay

The inhibition of α‐glucosidase activity was determined using the modified published method (Elya et al., 2012). 10 mM *p*NPG solution (400 μl), 400 μl PBS buffer, and 100 μl plant extract (10 mg/ml in dimethylsulfoxide, DMSO) were preincubated for 3 min at 37°C. After preincubation, 100 μl glucosidase enzyme (0.15 U/ml) was added to all and incubated for 15 min. The reaction was terminated by the addition of 2000 μl Na_2_CO_3_ (200 mM). The absorbance of the mixture at 405 nm was measured. Acarbose was used as the control/standard inhibitor

The percentage lipase inhibition was calculated by the following formula;

$$\%\;\text{Glucosidase inhibition activity}=\frac{\text{Absorbance of Control}-\text{Absorbance of test}}{\text{Absorbance of Control}}\times 100$$

### 2.6 Determination of the heat stability of the inhibitory effect

The extracts were dissolved in the corresponding buffer, and the solutions were boiled for 20 min in a boiling water bath at 100°C. Then, the extract solutions were cooled, and volumes were adjusted to original volumes with buffer and assayed for lipase or amylase or glucosidase inhibitory activities.

### 2.7 2,2‐Diphenyl‐1‐picrylhydrazyl (DPPH) radical scavenging assay

DPPH solution (1.8 ml) of 0.004% was mixed with 200 μl extract solution (10 mg/ml in methanol) to get a final concentration of 1 mg/ml. These solution mixtures were kept in the dark for 30 min, and absorbance was measured at 517 nm. The absorbance was recorded and % scavenging activity was calculated (Tepe, Eminagaoglu, Akpulat, & Aydin, 2005). BHA (1 mg/ml) was used as the control/standard for the assay.

$$\%\;\text{Scavenging activity}=\frac{\text{Absorbance of Control}-\text{Absorbance of test}}{\text{Absorbance of Control}}\times 100$$

The extracts were boiled in a water bath at 100°C for 20 min. Then, the extract solutions were cooled, and volumes were adjusted to original volumes with buffer and assayed for the antioxidant activity by conducting the DPPH radical scavenging assay.

### 2.8 Statistical analysis

All experiments were performed in three different sets, with each set in triplicates. Results were expressed as the mean ± *SD* of n experiments. The data were analyzed by repeated measurements of one‐way analysis of variance (ANOVA) using Minitab version 17. Differences between means were considered significant if *p* < 0.05 as denoted in applicable tables, figures, and the text.

## 3 RESULTS

### 3.1 Yield of plant extracts

The yields of crude methanol extracts obtained from 100 g of each plant material from the spices studied are tabulated in Table 1. The highest yield (8.4%) was obtained from the crude methanol extract of *T. foenum‐graecum*.

### 3.2 Optimization of lipase inhibition assay

**Figure 1:**
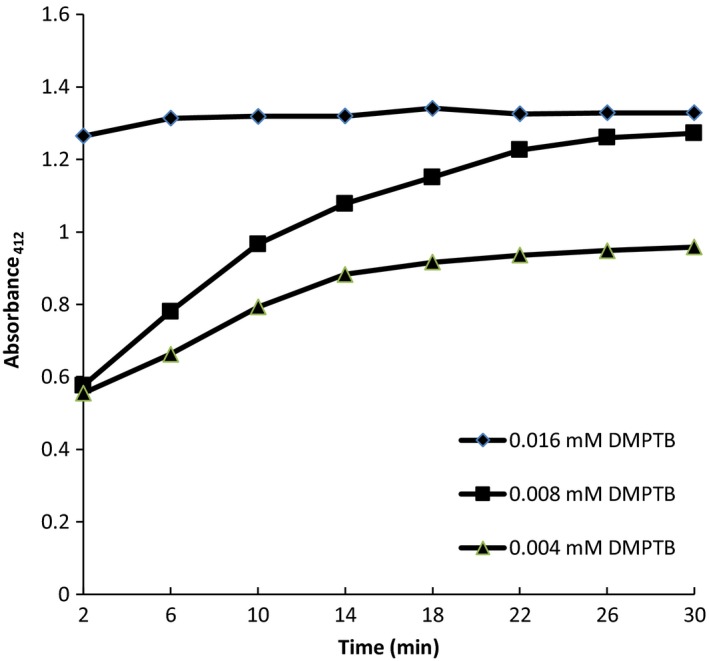
Linearity evaluation. Reaction mixtures were prepared with different amounts (0.004, 0.008, and 0.016 mM) of 2,3‐dimercapto‐1‐propanol tributyrate (DMPTB) and incubated with porcine pancreatic lipase at 37°C. The absorbances were recorded for 30 min at 412 nm

The lipase concentration employed in the assay was restricted to a maximum of 8U/ml due to the lack of solubility of lyophilized powder of lipase at higher concentrations. As shown in Figure 1, the initial reaction rate increased with the increasing concentration of the substrate. The reaction mixture that contained 0.008 mM DMPTB showed a linear absorbance change up to 22 min, with absorbance reaching a plateau above 1.326 absorbance unit after further incubation (Figure 1), whereas the reaction mixture that contained 0.004 mM DMPTB showed a linear absorbance change only up to 10 min, reaching a plateau above 0.793 absorbance unit. But the reaction mixture that contained 0.016 mM DMPTB showed no linear absorbance change (Figure 1).

### 3.3 Inhibition of lipase activity

Among the unboiled extracts tested, the crude methanol extract of *Trigonella foenum‐graecum* was the most effective inhibitor of pancreatic lipase activity, while *M. fragrans, C. cyminum, E. cardamomum*, and *C. sativum* also showed an inhibitory activity (Table 2). The extract from *S. aromaticum* did not inhibit pancreatic lipase, whereas *C. longa, F. officinalis*, and *C. zeylanicum* crude methanol extracts showed an activation of the lipase enzyme (Table 2).

**Table 2:** Percentage lipase inhibitory activity of unboiled and boiled crude methanol extracts of spices

Plant species	% Inhibition activity unboiled	% Inhibition activity boiled
*Brassica juncea*	8.80 ± 1.56	6.96 ± 1.76
*Cinnamomum zeylanicum*	−16.98 ± 1.34	8.47 ± 1.43^a^
*Coriandrum sativum*	6.19 ± 1.15	4.03 ± 1.21
*Cuminum cyminum*	19.17 ± 1.54	12.57 ± 1.87^a^
*Curcuma longa*	−8.4 ± 1.54	−6.79 ± 1.34
*Elettaria cardamomum*	11.76 ± 1.55	8.74 ± 1.54
*Foeniculum officinale*	−16.05 ± 1.63	9.54 ± 1.31^a^
*Myristica fragrans*	22.7 ± 1.45	15.74 ± 1.44^a^
*Syzygium aromaticum*	0.12 ± 1.13	0.15 ± 1.12
*Trigonella foenum‐graecum*	25.42 ± 1.32	20.23 ± 1.78^a^
Orlistat	98.8 ± 0.91	NA

*Notes*

2,3‐Dimercapto‐1‐propanol tributyrate (DMPTB) was used as the substrate, and the final concentration of the crude extracts was at 1 mg/ml. The amount of thiol released was measured after the incubation for 6 mins at 37°C with 412 nm. Orlistat is taken as standard inhibitor. Results were presented as mean ± standard deviation, and mean was taken as the average of three readings of three different experiments. “‐” indicates a promotion of pancreatic lipase activity.

NA, not applicable.

a The inhibitory activity in boiled extract is significantly (*p *<* *0.05) different to the corresponding unboiled extract.

All the boiled extracts exhibited a pancreatic lipase inhibitory activity (Table 2) except crude methanol extract of *C. longa*. The pancreatic lipase inhibitory activities of the boiled extracts of *C. cyminum*,* M. fragans*, and *T. foenum‐graecum* were significantly (*p *<* *0.05) lower than their unboiled extracts*. *The unboiled methanol extracts of *C. zeylanicum* and *F. officinale* showed an activation of pancreatic lipase enzyme but boiling these extracts significantly (*p *<* *0.05) changed the activity into an inhibition of the lipase enzyme (Table 2).

### 3.4 Inhibition of amylase activity

All ten unboiled crude methanol extracts were subjected to the amylase inhibition assay, using the preincubation method and only six crude extracts exhibited inhibition activity (Table 3). In those extracts, the pancreatic amylase inhibitory activity was in the following order, from the highest to the lowest: *S. aromaticum *> *C. longa *> *C. zeylanicum* > *F. officinale* > *B. juncea* > *T. foenum‐graecum* (Table 3). Other four unboiled extracts showed an activation of the pancreatic amylase enzyme (Table 3).

**Table 3:** Percentage amylase inhibitory activity of unboiled and boiled crude methanol extracts of spices

Plant species	% Inhibition activity unboiled	% Inhibition activity boiled
*Brassica juncea*	20.1 ± 1.50	16.6 ± 1.67
*Cinnamomum zeylanicum*	32.39 ± 1.91	28.59 ± 1.78
*Coriandrum sativum*	−33.33 ± 1.43	−23.98 ± 1.27^a^
*Cuminum cyminum*	−7.14 ± 1.12	1.16 ± 1.14^a^
*Curcuma longa*	52.2 ± 1.65	47.78 ± 1.98^a^
*Elettaria cardamomum*	−33.33 ± 1.67	−26.89 ± 1.67^a^
*Foeniculum officinale*	28.79 ± 1.22	26.73 ± 1.75
*Myristica fragrans*	−5.81 ± 1.45	−5.74 ± 1.44
*Syzygium aromaticum*	58.10 ± 1.24	52.82 ± 1.45^a^
*Trigonella foenum‐graecum*	8.69 ± 1.35	7.78 ± 1.19
Acarbose	87.67 ± 1.76	NA

*Notes*

Preincubation chromogenic method from Geethalakshmi et al. (2010) was adapted, and the final concentrations of the crude extracts were 1 mg/ml. The amylase inhibition was analyzed by amount of maltose production from starch at 517 nm after incubation at 37°C. Acarbose was used as the standard inhibitor. Results were presented as mean ± standard deviation, and mean was taken as the average of three readings of three different experiments. “‐” indicates a promotion of pancreatic amylase activity.

NA, not applicable.

a The inhibitory activity in boiled extract is significantly (*p *<* *0.05) different to the corresponding unboiled extract.

All the unboiled crude extracts which showed an amylase inhibitory activity exhibited a lower inhibitory activity in the boiled form (Table 3). Among them, the boiled extracts of *S. aromaticum* and *C. longa* showed a significantly (*p *<* *0.05) lower inhibitory activity (Table 3). Further, the unboiled extract of *C.cyminum*, which exhibited an activation of amylase enzyme, inhibited the enzyme in the boiled form (Table 3). The other three boiled extracts were showing activation of the pancreatic amylase enzyme (Table 3), whereas *C. sativum* and *E. cardamomum* extracts showed a significant (*p *<* *0.05) reduction in the enzyme activation in the boiled form.

### 3.5 Inhibition of glucosidase activity

Among the seven unboiled crude methanol extracts with glucosidase inhibitory activity, *C. zeylanicum*,* M. fragans*, and *S. aromaticum* had the strongest effect on inhibiting α‐glucosidase enzyme from *Saccharomyces cerevisiae* (Table 4). Unboiled crude extract of *C. zeylanicum* showed the highest α‐glucosidase inhibition of 96.78 ± 0.45% at 1 mg/ml. Unboiled extracts of *C. sativum*,* C. longa*, and *T. foenum‐graecum* showed a mild inhibition of the glucosidase enzyme (Table 4). Unboiled extracts of *B. juncea, C.cyminum*, and *F.officinale* showed neither activation nor inhibition of the α‐glucosidase enzyme (Table 4).

**Table 4:** Percentage glucosidase inhibitory activity of unboiled and boiled crude methanol extracts of spices

Plant species	% Inhibition activity unboiled	% Inhibition activity boiled
*Brassica juncea*	0.0	0.0
*Cinnamomum zeylanicum*	96.78 ± 12.45	95.64 ± 1.47
*Coriandrum sativum*	2.58 ± 1.76	0.0
*Cuminum cyminum*	0.0	0.0
*Curcuma longa*	3.72 ± 1.62	1.78 ± 0.98
*Elettaria cardamomum*	22.87 ± 1.98	21.90 ± 1.01
*Foeniculum officinale*	0.0	0.0
*Myristica fragrans*	90.64 ± 1.23	89.65 ± 1.31
*Syzygium aromaticum*	88.91 ± 1.12	86.89 ± 1.98
*Trigonella foenum‐graecum*	3.2 ± 1.76	2.54 ± 1.21
Acarbose	99.52 ± 0.54	NA

*Notes*

*p*‐nitrophenyl‐α‐D‐glucopyranoside was used as the substrate, and the final concentrations of the crude extracts were at 1 mg/ml. The amount of *p*‐nitro phenol released was measured after the incubation for 15 mins at 37°C with 405 nm. Acarbose was used as the standard inhibitor. Results were presented as mean ± standard deviation, and mean was taken as the average of three readings of three different experiments.

NA, not applicable.

Boiled extracts of six spices exhibited a glucosidase inhibitory activity, and those activities were not significantly (*p *<* *0.05) lower than the unboiled extracts (Table 4). Meanwhile, the boiled extracts of *B. juncea, C. sativum, C. cyminum*, and *F. officinale* showed neither activation nor inhibition of the α‐glucosidase enzyme.

### 3.6 Antioxidant activity

All the ten unboiled and boiled extracts of spices had pronounced radical scavenging antioxidant activity (Table 5). The antioxidant activity of the unboiled extracts of *C sativum*,* C. cyminum*, and *E. cardamomum* was 94.80%, 92.92%, and 91.61%, respectively, at 1 mg/ml and were statistically similar (*p *<* *0.05) to that of BHA (94.97%).

**Table 5:** Percentage antioxidant activity of unboiled and boiled crude methanol extracts of spices

Plant species	% Scavenging activity unboiled	% Scavenging activity boiled
*Brassica juncea*	59.01 ± 1.10	61.23 ± 1.54
*Cinnamomum zeylanicum*	83.41 ± 1.30	76.97 ± 1.89^a^
*Coriandrum sativum*	94.80 ± 1.15	96.56 ± 1.12
*Cuminum cyminum*	92.92 ± 1.13	93.43 ± 1.34
*Curcuma longa*	76.55 ± 1.13	71.45 ± 1.23^a^
*Elettaria cardamomum*	91.61 ± 1.32	93.87 ± 1.15
*Foeniculum officinale*	90.11 ± 1.22	91.23 ± 1.51
*Myristica fragrans*	83.41 ± 1.21	89.76 ± 1.56^a^
*Syzygium aromaticum*	89.25 ± 1.24	90.53 ± 1.23
*Trigonella foenum‐graecum*	90.76 ± 1.22	84.67 ± 1.13^a^
BHA	94.97 ± 1.01	NA

*Notes*

2,2‐Diphenyl‐1‐picrylhydrazyl (DPPH) radical scavenging assay, by measuring the absorbance at 517 nm after incubation for 30 min in the dark. The final concentrations of the crude extracts were at 1 mg/ml. BHA was used as the standard. Results were presented as mean ± standard deviation, and mean was taken as the average of three readings of three different experiments.

NA, not applicable.

a The inhibitory activity in boiled extract is significantly (*p *<* *0.05) different to the corresponding unboiled extract.

The boiled extracts of *B. juncea, C. sativum, C. cyminum, E.cardamomum, F. officinale, M. fragans*,* and S. aromaticum* had a higher antioxidant activity than the unboiled extracts. However, only the *M. fragans* showed a significantly (*p *<* *0.05) higher antioxidant activity. Alternatively, the boiled extracts of *C. zeylanicum, C. longa*, and *T. foenum‐graecum* had significantly (*p *<* *0.05) lower antioxidant activity than their unboiled extracts.

## 4 Discussion

Cooking represents an indispensable prerequisite in obtaining quality, palatability, and digestibility of food (Adefegha & Oboh, 2011). It is well known that thermal processing such as boiling, frying, and microwave cooking might be able to modulate the secondary metabolites present in plant materials (Kaur & Kapoor, 2001; Rohn, Buchner, Driemel, Rauser, & Kroh, 2007) and also may modulate the biological activity of plant secondary metabolites (Estbeyoglu, Ulbrich, Rehberg, Rogn, & Rambach, 2015). Studies conducted to investigate the thermal stability of secondary metabolites of spices are very scarce. In such studies, thermal processing was reported to cause reduction in flavonoid and polyphenol contents of spices based on the magnitude and extent of heat and duration of heating (Adefegha et al., 2016; Settharaksa, Jongjareonrak, Hmadhlu, Chansuwan, & Siripongvutikorn, 2012).

Despite the large number of research studies conducted to investigate inhibitory potential of spice crude extracts on lipase, amylase, and glucosidase enzymes, the current study is the first reported study revealing the heat stability of the lipase, amylase, and glucosidase inhibitors in crude spice extracts. The results of the current study revealed that unboiled spice extracts had a higher lipase and amylase inhibitory activities than boiled extracts. Major compounds present in spices such as apegenins in *T. foenum‐graecum* (seed) (Fernando, 2016), eugenol in *M. fragrans* and *S. aromaticum* (Mnafgui et al., 2013), and cucuminoids in *C. longa* (Lekshmi, Arimboor, Raghu, & Menon, 2012) have inhibited the digestive enzymes. Several researchers have identified changes in the concentration of these secondary metabolites due to thermal processing such as boiling (Baker, Chogan, & Opara, 2013; Tomaino et al., 2005), and this might be the reason for the change in the inhibitory activities between the boiled and the unboiled extracts observed in this study. Further, flavonoids in spices exist in free and conjugated forms and may breakdown by enzyme, acid, or heat treatment (Cartea, Francisco, Soengas, & Velasco, 2011), and change in the inhibitory activities of boiled and unboiled extracts may be due to the variation of the inhibitory activities in these two forms. However, recent literature data did not show a consistent trend for the effects of thermal processing on secondary metabolite contents in spices (Baker et al., 2013; Khatun, Eguchi, Yamaguchi, Takamura, & Matoba, 2006). This suggests that the effect of thermal processing on secondary metabolites varies in different spices and deserves further research. Therefore, the reason for the modulation in the inhibition of lipase, amylase, and glucosidase activities as a result of boiling of the spices could not be categorically stated.

Among crude extracts, *T. foenum‐graecum* (seed), *C. cyminum* (seed), and *M. fragrans* (seed) extracts showed lipase inhibitory activity in unboiled and boiled forms. Further, the unboiled form of these extracts showed a significantly higher inhibitory activity than the boiled form, and this suggested the extraction procedures which do not involve heating to be more effective than the extraction procedures which involve heating in extracting lipase inhibitors from these spices. Unboiled extracts of *T. foenum‐graecum* (seed), *C. cyminum* (seed), and *M. fragrans* (seed) had more potential in inhibiting the major lipid digesting enzyme pancreatic lipase. Therefore, these extracts may have more potential as dietary therapy in controlling obesity and dyslipidemias.

In Asian countries, a drink prepared from *T. foenum‐graecum* (seed) is consumed as an infusion or as a decoction. Therefore, considering results from the current study, the use of *T. foenum‐graecum* (seed) extract in the form of infusion could be recommended to be more beneficial than the decoction in controlling obesity and dyslipidemias. In the past, seeds of *M. fragrans* were added to puddings as a flavoring agent but, at present, this practice has been discontinued in Sri Lanka. This study shows the hypolipidemic potential of *M. fragrans* seeds in the boiled form and endorses the need of resuscitation of the old culinary practice.

Research has revealed that stronger inhibition of α‐glucosidase activity and mild inhibition of α‐amylase activity of drugs/extracts could address the major drawbacks of currently used synthetic α‐glucosidase and α‐amylase inhibitors (Bischoff, 1994). The current study shows the potential of unboiled and the boiled forms of *M. fragrans* seed to address this issue, and therefore, *M. fragrans* seed could be beneficial in the management of postprandial hyperglycemia in diabetic patients and healthy individuals.

Antioxidants are molecules that slow down or prevent oxidation of biomolecules. Antioxidant activity was increased in *E.cardamomum, M. fragans*,* and S. aromaticum* boiled extracts in the present study and in support of this finding, Chan et al. (2015), Baker et al. (2013), and Tomaino et al. (2005) have reported increased antioxidant activity in *E.cardamomum, M. fragans*,* and S. aromaticum* even after boiling, suggesting that the active components are relatively stable during thermal treatment. Large numbers of studies that have been conducted to evaluate the effect of temperature on the antioxidant activity of spices could not be compared with the present study because those studies used different solvent systems and/or different extraction procedures (Khatun et al., 2006).

## 5 CONCLUSIONS

Boiling the spices modulated the inhibitory potentials for lipase, amylase, and glucosidase enzymes that are already present in the spices. Conducting in vivo studies using unboiled and boiled extracts to prove the hypothesis developed by the in vitro studies is beneficial. Overall, the properties investigated in this study will be of value in the use of spices as antiobesity and antidiabetic agents.

## CONFLICT OF INTEREST

The authors declare that they have no competing interests.

## ETHICAL APPROVAL AND CONSENT TO PARTICIPATE

Ethics approval and consent to participate is not applicable to this manuscript, since it does not report on or involve the use of any animal or human data or tissues.

## CONSENT FOR PUBLICATION

Not applicable.
